# Combination Treatment with Hydroxytyrosol and Vitamin E Improves NAFLD-Related Fibrosis

**DOI:** 10.3390/nu14183791

**Published:** 2022-09-14

**Authors:** Nadia Panera, Maria Rita Braghini, Annalisa Crudele, Antonella Smeriglio, Marzia Bianchi, Angelo Giuseppe Condorelli, Rebecca Nobili, Libenzio Adrian Conti, Cristiano De Stefanis, Gessica Lioci, Fabio Gurrado, Donatella Comparcola, Antonella Mosca, Maria Rita Sartorelli, Vittorio Scoppola, Gianluca Svegliati-Baroni, Domenico Trombetta, Anna Alisi

**Affiliations:** 1 Research Unit of Molecular Genetics of Complex Phenotypes, Bambino Gesù Children’s Hospital, IRCCS, 00146 Rome, Italy; 2 Department of Chemical, Biological, Pharmaceutical and Environmental Sciences, University of Messina, Viale Ferdinando Stagno d’Alcontres 31, 98166 Messina, Italy; 3 Genodermatosis Unit, Genetics and Rare Diseases Research Division, Bambino Gesù Children’s Hospital, IRCCS, 00146 Rome, Italy; 4 Core Facilities, Bambino Gesù Children’s Hospital, IRCCS, 00146 Rome, Italy; 5 Department of Gastroenterology, Polytechnic University of Marche, 60121 Ancona, Italy; 6 Unit of Hepatology, Gastroenterology, and Nutrition, Bambino Gesù Children’s Hospital, IRCCS, 00165 Rome, Italy; 7 Liver Injury and Transplant Unit, Polytechnic University of Marche, 60121 Ancona, Italy; 8 Obesità Center, Polytechnic University of Marche, 60121 Ancona, Italy

**Keywords:** NAFLD, fibrosis, antioxidants, PIIINP, NOX2

## Abstract

Non-alcoholic fatty liver disease (NAFLD)-related liver fibrosis results in the encapsulation of injured liver parenchyma by a collagenous scar mainly imputable to hepatic stellate cells’ activation. Approved pharmacological treatments against NAFLD-related fibrosis are still lacking, but natural compounds such as hydroxytyrosol (HXT) and vitamin E (VitE), are emerging as promising therapeutic opportunities. In this study, the potential anti-fibrotic effect of HXT + VitE combination therapy was investigated in vitro and in vivo. In particular, tumor growth factor (TGF)-β-activated LX-2 cells as an in vitro model, and carbon tetrachloride plus a Western diet as a mice model were employed. The effect of HXT + VitE on fibrosis was also investigated in children with biopsy-proven NAFLD. Our results demonstrated that HXT + VitE caused a reduction of proliferation, migration, contractility, and expression of pro-fibrogenic genes in TGF-β-activated LX-2 cells. HXT + VitE treatment also antagonized TGF-β-dependent upregulation of pro-oxidant NOX2 by interfering with nuclear translocation/activation of SMAD2/3 transcription factors. The mouse model of NAFLD-related fibrosis treated with HXT + VitE showed a marked reduction of fibrosis pattern by histology and gene expression. Accordingly, in children with NAFLD, HXT + VitE treatment caused a decrease of circulating levels of PIIINP and NOX2 that was supported over time. Our study suggests that HXT + VitE supplementation may improve NAFLD-related fibrosis.

## 1. Introduction

Non-alcoholic fatty liver disease (NAFLD), which is defined as intrahepatic accumulation of fat in more than 5% of hepatocytes, and in the absence of other conditions such as alcohol consumption, drug abuse, viral hepatitis infections, or genetic and autoimmune diseases, is now considered the most common chronic liver disease in both adults and children [1,2,3].

NAFLD comprises a broad spectrum of liver lesions ranging from isolated steatosis to the multi-faced non-alcoholic steatohepatitis (NASH) characterized by ballooning, inflammation, and eventually fibrosis, which may evolve to cirrhosis and hepatocellular carcinoma [4]. The histopathological heterogeneity of this disease may reveal which pattern may progress to a more severe disease (e.g., fibrosis and cirrhosis).

During the last two decades, the increasing rate of obesity prevalence in the child population has contributed to the parallel rising of NAFLD prevalence in children and adolescents worldwide, affecting approximately 7.6% of the general pediatric population and reaching almost 34% in obese pediatric subjects [5]. Histology-based studies have estimated the prevalence rate of NASH between 20% and 50%, and advanced fibrosis between 10% and 20% in children with NAFLD [6]. Among these children, only a few cases of NAFLD may rapidly progress toward cirrhosis; the progression to hepatocellular carcinoma is a rare event reported only one pediatric case. Therefore, currently liver transplantation in children with NAFLD is indicated only if the disease progresses to the end-stage liver disease at a pediatric age [7]. Nevertheless, the retrospective analysis of a cohort reported that NASH is becoming the most common cause of liver transplant in U.S. young adults [8].

Despite the poor knowledge about the natural history of pediatric NAFLD, current literature suggests that the disease in children can progress rapidly. The rapid progression of NAFLD in children could be ascribable to the combination of multiple parental, in utero, or post-natal factors. Indeed, most of the heritability of pediatric NAFLD could depend on genetic and epigenetic factors, such as predisposing gene variants and preconception parental nutritional status, respectively [9,10,11].

In this scenario, the hereditability of NAFLD represents a breeding ground for the pathogenic effect of additional drivers, including lifestyle habits and nutrition which may lead to the progression toward fibrosis in childhood [12].

Because there are no Food and Drug Administration (FDA)-approved treatments for pediatric NAFLD and its progressive form, current therapeutic protocols for overweight/obese children affected by this disease are centered on physical activity and diet, mainly accompanied by biologically based complementary treatment approaches (e.g., vitamins, omega-3, and probiotics) [13,14]. Recently, polyphenols are emerging as promising natural compounds to counteract the metabolic changes and cellular derangements occurring during NAFLD in preclinical and clinical studies [15]. In particular, the administration of extra virgin olive oil (EVOO)-derived polyphenols has been associated with the improvement of steatosis, inflammation, and liver damage as documented by in vivo and in vitro models [16]. One interesting phenolic component of EVOO is hydroxytyrosol (HXT) which provides health benefits in animal models when combined with other natural supplementations such as omega-3 and vitamin E (VitE) [17]. Despite these findings, the beneficial effects of HXT in human NAFLD are still poor. Indeed, we found that the daily intake of a combined supplementation of HXT and VitE improved hepatic steatosis, oxidative stress, and systemic inflammation in pediatric NAFLD [18,19]. However, whether this compound is also able to attack fibrosis has to be defined.

Hence, the present study attempts to investigate the anti-fibrotic properties of a combination therapy with HXT and VitE in both in in vitro and in vivo models, as well as in pediatric NAFLD.

## 2. Materials and Methods

### 2.1. Cells, Treatments, and Cell Viability

Human hepatic stellate cells (HSCs) LX-2 were maintained in Dulbecco’s Modified Eagle Medium (DMEM) supplemented with 10% fetal bovine serum (FBS) and 1% penicillin and streptomycin (Thermo Fisher Scientific-Gibco, Waltham, MA, USA) at 37 °C in a humidified atmosphere of 5% CO_2_ and 95% air. Cells were screened for possible mycoplasma contamination by using Venor GeM Advance Mycoplasma Detection KIT (Minerva Biolabs GmbH, Berlin, Germany). All the experiments were performed only in mycoplasma-free cells.

Before the experiments, LX-2 cells were pre-cultured in DMEM supplemented with 1% FBS for 12 h. Next, cells were treated with different concentrations of HXT (20, 30, 40, and 50 µM), or VitE (10, 20, 50, and 100 µM), or nothing for 24 or 48 h to evaluate the effect of compounds on cell viability. Cell viability at the different timepoints was evaluated by using the cell proliferation kit II-XTT (Merck-Roche, Darmstadt, Germany) according to the manufacturer’s protocol. The adsorbance/optical density (OD) was measured at 492 and 620 nm by using a spectrophotometer.

For the next experiments, LX-2 cells remained untreated or were activated with 10 ng/mL tumor growth factor (TGF)-β for 24 h (Thermo Fisher Scientific-PeproTech, Waltham, MA, USA), followed by selected conditions of single, HXT, or VitE, or combined (HXT + VitE) treatments for a further 24 h. Both HXT and VitE for the in vitro study were purchased from Selleck Chemicals (Houston, TX, USA).

### 2.2. Cell Proliferation Assay

LX-2 cells were seeded at a density of 5 × 10^3^ cells per well in 96-well plates in 100 μL 1% FBS supplemented DMEM and incubated at 37 °C overnight. Subsequently, cells were pretreated or not with TGF-β (10 ng/mL) for 24 h and then exposed to HXT (30 µM) and VitE (20 µM) alone or as a combined treatment for a further 24 h. A DELFIA^®^ cell proliferation kit (PerkinElmer, Waltham, MA, USA) was used to determine the changes in the proliferation rate between different experimental conditions. As described in the assay protocol, the amount of BrdU incorporated in DNA was determined by a europium-labeled antibody. The dissociation of europium ions from the anti-BrdU antibody and the production of fluorescent and stable chelates was obtained by a DELFIA inducer reagent. The fluorescent signal, which correlates with the amount of DNA synthesis, was measured by a time-resolved fluorometer 2100 EnvisionTM multilabel reader (PerkinElmer Waltham, MA, USA).

### 2.3. Real-Time Monitoring of Cell Proliferation

Real-time cell proliferation changes were measured by using an Incucyte^®^ label-free cell proliferation assay (Sartorius-Biopharma, Göttingen, Germany). LX-2 cells were seeded in quintuplicate at a density of 2 × 10^3^ cells per well in 96-well plates in 100 μL of 1% FBS supplemented with DMEM and incubated at 37 °C overnight. Subsequently, cells were pretreated or not with TGF-β (10 ng/mL) for 24 h and then exposed to HXT (30 µM) and VitE (20 µM) alone or as a combined treatment. Next, cells were placed into the Incucyte^®^ live-cell analysis system (Sartorius-Biopharma, Göttingen, Germany). The time-lapse imaging has been set to acquire four images per well from five technical replicates every 3 h by using a 10× objective lens over a time course of 24 h, and then classic confluence analysis was performed by using IncuCyte™ basic software.

### 2.4. Quantitative Real-Time Polymerase Chain Reaction (qRT-PCR)

qRT-PCR was performed as previously described to determine the gene expression levels in the cultured cells [20]. Briefly, LX-2 cells were seeded in 6-well cell culture plates at a density of 1 × 10^5^ cells/well and cultured in 1% FBS supplemented with DMEM overnight. After media removal, cells were treated or not with 10 ng/mL TGF-β for 24 h, and then exposed to single or coupled treatment with 30 µM HXT and 20 µM VitE for 24 h. Next, the media was removed, and the cells were rinsed three times with cold phosphate-buffered saline (PBS). Total RNA was isolated from the cells by using the total RNA purification plus kit (Norgen Biotek Corp, Thorold, ON, Canada). Genomic DNA was digested by using genomic DNA removal (Norgen Biotek Corp, Thorold, ON, Canada). The mRNA level expression of target genes was determined by using specific TaqMan commercial probes by Thermo Fisher Scientific-Applied Biosystems (Waltham, MA, USA): these included α-smooth muscle actin-α-SMA (ACTA2 Hs.00909449_m1), collagen type 1 (COL1A1 Hs00164004_m1), collagen type 3 (COL3A1 Hs00943809_m1), and Gp91-containing NADPH oxidases (NOX2 Hs00166163_m1). The mRNA levels were normalized to endogenous control gene glyceraldehyde 3-phosphate dehydrogenase (GAPDH Hs02786624_g1). The gene expression levels were represented as fold changes versus control and calculated by the ΔΔCt method.

### 2.5. Immunofluorescence (IF)

For IF, LX-2 cells were seeded at a density of 2 × 10^4^ cells/well in a 4-well chamber slide (Nunc, Naperville, IL, USA) incubated overnight in 1% FBS supplemented with DMEM at 37 °C and treated as described for qRT-PCR. (Section 2.4) Cells were fixed in 4% paraformaldehyde in PBS and permeabilized in PBS Triton X-100 for 15 min. The cells were incubated with PBS containing 5% bovine serum albumin (BSA) for 1 h at room temperature (RT) to block non-specific interactions. The 5% BSA (PBS) solution was used for all incubations and antibody dilutions. For phalloidin staining, rhodamine phalloidin (Thermo Fisher Scientific-Invitrogen, Waltham, MA, USA) was diluted 1:100 in 5% BSA (PBS) solution. The solution was applied on each sample and incubated for 20 min at RT in the dark. Rabbit anti-Mothers Against Decapentaplegic 2 (SMAD)2/3 antibody (1:100 dilution) (Cell Signaling Technology, MA, USA) was incubated overnight at 4 °C. The antibody was visualized with the anti-rabbit Alexa Fluor^®^ 488 (1:500 dilution) (Thermo Fisher Scientific-Invitrogen, Waltham, MA, USA) for 1 h at RT. Cell nuclei were blue stained with Hoechst 33342 (Life Technologies-Invitrogen, Carlsbad, CA, USA). The images of the immunostained cells were acquired by a white light laser confocal microscope TCS SP8 (Leica Corporation, Wetzlar, Germany).

### 2.6. Migration Assay

Migration was evaluated by seeding LX-2 cells in a 2-well silicone insert with a defined cell-free gap at 5 × 10^4^ cells per silicone well in 1% FBS containing DMEM in 12-well culture plates (ibidi, Gräfelfing, Germany) in order to suppress serum-driven proliferation effects. Next, inserts were carefully removed to generate a cell-free zone, and images of the initial gap area were captured at four positions by inverted microscope (10×, Olympus 1X71). The cells were then rinsed with PBS three times and incubated in the presence or absence of 10 ng/mL TGF-β alone or in combination with HXT + VitE. After treatment, the cells were followed up to 12 h, when images of final migration were taken. Cell migration was determined by recording the movement of cells into the scraped area, and quantitative analysis was performed by ImageJ software (version 1.52a, National Institutes of Health, Bethesda, MD, USA).

### 2.7. Collagen Lattice Contraction Assay

Cell contractility was evaluated by using a collagen lattice contraction assay as previously described with minor modifications [21]. Collagen solution was prepared by mixing acidic-soluble type I collagen from calf hides (Symatese, Chaponost, France), a 5-fold concentration of DMEM, and a buffer solution (0.05 M NaOH, 2.2% NaHCO_3_, 200 mM HEPES) in the ratio 7:2:1, *w*/*w*/*v*. The final concentration of type I collagen was 2.1 mg/mL. One milliliter of collagen solution was poured into 12-well culture plates and incubated for 1 h at 37 °C to allow gelation. Subsequently, LX-2 cells were suspended in DMEM supplemented with 10% FBS and plated on the collagen gels (5 × 10^5^ cells/well). After 12 h of incubation, gels were detached from each well and left floating. Then HXT + VitE in the presence or absence of 10 ng/mL TGF-β, were added into each well. The diameters of the collagen lattices were monitored for 24 h after stimulant addition. Images were acquired with ChemiDocTM XRS+ System (Bio-Rad, Hercules, CA, USA) at 24 h. The surface area of gel samples was calculated by employing ImageJ software (version 1.52a, https://imagej.nih.gov/ij/download.html (accessed on 15 December 2021). The contraction area was defined as the percentage of the difference of reduced collagen gel surface area after collagen lattice release. The results are based on triplicate experiments.

### 2.8. Intracellular Reactive Oxygen Species (ROS)

Intracellular ROS levels in LX-2 cells were evaluated by CM-H_2_DCFDA assay based on a chloromethyl derivative of H_2_DCFDA (cell-permeant probe 2’,7’-dichlorodihydrofluorescein diacetate). Briefly, cells were seeded into a 96-well black plate (8 × 10^3^ cell/well) and after 24 h of TGF-β pretreatment and further 24 h exposure to HXT and VitE treatment, cells were incubated with Hanks’ balanced salt solution (HBSS) buffer containing the fluorescent probe at a final working concentration of 10 μM for 30 min at 37 °C. Blank wells (with non-stained cells) were used as a control. The fluorescence intensity was measured at 495 nm excitation and 530 nm emission by using a BioTek Synergy H1 microplate reader (Agilent, Santa Clara, CA, USA), and the values were normalized for cell amounts by nuclear staining with Hoechst 33342 (Life Technologies-Invitrogen).

### 2.9. Western Blot (WB)

LX-2 cells were plated in T75 tissue culture flasks in complete medium and maintained in 1% FBS DMEM overnight and then treated as described for qRT-PCR PCR (Section 2.4) Cells were harvested by using a cell scraper and cold PBS and lysed in RIPA buffer (Merck-Sigma-Aldrich) containning alt protease and phosphate inhibitor cocktail (100×) (Thermo Fisher Scientific-Pierce, Waltham, MA, USA). After extraction, the total proteins were measured by using a BCA assay kit by Thermo Fisher Scientific-Pierce. Equal amounts of protein samples (30 μg of protein per lane) were loaded and resolved onto a 10% Bolt Bis-Tris plus mini gel. The iBlot^®^ 2 gel transfer stacks mini integrated with nitrocellulose transfer membranes were used to transfer protein by using the dry blotting iBlot 2 gel transfer device (all provided by Thermo Fisher Scientific-Invitrogen, Waltham, MA, USA). The membranes were blocked with 5% BSA, then incubated with primary antibodies overnight at 4 °C and incubated with the appropriate secondary antibodies. Primary antibodies used were: rabbit anti-NOX2 antibody (1:100 dilution; 19013-1-AP) (Proteintech Group Inc., Rosemont, IL, USA) and rabbit anti-GAPDH antibody (1:5000 dilution; 5174) (Cell Signaling Technology Inc., Danvers, MA, USA). Detection was achieved by HRP-conjugated anti-rabbit (1:10,000 dilution) (Jackson ImmunoResearch, Ely, Cambridgeshire, UK). Immunoreactive bands were detected by enhanced chemiluminescence with clarity Western ECL substrate (Bio-Rad Laboratories Inc., Hercules, CA, USA) and images were captured by iBright imaging systems (Thermo Fisher Scientific-Invitrogen). Protein expression was quantified by densitometric analysis by using ImageJ software (version 1.52a).

### 2.10. Pilot Animal Study

Ten eight-week-old male FVB/N mice were fed with Western diet (WD) containing 21.2% fat, 48.5% carbohydrates, and 17.3% proteins by weight (TD. 120528, by Envigo, Bresso, Italy). Contextually, a high sugar solution containing 23.1 g/L d-fructose and 18.9 g/L d-glucose (Merck-Sigma-Aldrich, Darmstadt, Germany), in addition to an intraperitoneal injection of carbon tetrachloride-CCl_4_ (Merck-Sigma-Aldrich) at the dose of 0.2 μL/g of body weight, were administered once a week for 12 weeks. After this, the CCl_4_ treatment was stopped and mice were divided in two groups as follows: WD-fed animals receiving vehicle (*n* = 5), and WD-fed animals treated by gavage with 7.5 mg/kg HXT and 10 mg/kg VitE for 2 weeks (*n* = 5). All procedures involving animals and their care were approved by the Institutional Committee on the Ethics of Animal Experiments of the Marche Polytechnic University (Ancona, Italy), and conducted in conformity with EU Directive 2010/63/EU for animal experiments.

Liver samples were fixed with 10% formalin for 24 h and the dehydrated liver samples were then embedded in paraffin. Hematoxylin and eosin (H&E) and Masson’s trichrome staining were used to evaluate liver damage and fibrosis.

For the murine tissues, liver samples were sectioned into small pieces (approximately 2 mg) and homogenized in lysis solution provided in a Norgen’s total RNA isolation plus kit (Norgen Biotek Corp, Thorold ON, Canada) according to the manufacturer’s instructions. qRT-PCR analysis was performed as described above (Section 2.4). The mRNA level expression of target genes was determined by using the specific mouse TaqMan commercial probes (Thermo Fisher Scientific-Applied Biosystems, Waltham, MA, USA): α-SMA (Mm00725412_s1), COL1A1 (Mm00801666_g1), COL3A1 (Mm00802300_m1), NOX2 (Mm01287743_m1). The mRNA levels were normalized versus the endogenous controls GAPDH (Mm99999915_g1).

### 2.11. Study Patients

In this study, a subgroup of patients, who concluded a previous randomized, double-blind placebo-controlled trial that evaluated the effects of 4 months of treatment with HXT + VitE (protocol number: 1067_OPBG), was included [18]. In particular, this subgroup included all the patients that adhered to a second follow-up study of 24 months after the end of treatments (protocol number: 2055_OPBG).

The study population was composed of 9 patients in the Placebo (PLA) arm and 16 in the treatment arm (HXT + VitE). For details on treatments see the previous studies [18,19].

Liver biopsy data on fibrosis of the study population were available only at the baseline. Anthropometrical and biochemical parameters, and liver echography data were collected at baseline (T0), after 4 months of treatment (T1), and after 24 months from the end of treatment following procedures already described [18]. Total cholesterol, high-density lipoprotein (HDL)-cholesterol, low-density lipoprotein (LDL)-cholesterol, triglycerides, glucose, insulin, high-sensitivity C-reactive protein (hs-CRP), alanine aminotransferase (ALT), aspartate aminotransferase (AST), gamma-glutamyl transferase (GGT), uric acid, blood urea nitrogen, and creatinine were measured in all children by using standard laboratory procedures.

Homeostatic model assessment for IR (HOMA-IR) was also evaluated.

### 2.12. Enzyme-Linked ImmunoSorbent Assay (ELISA) 

For assessment of plasma N-terminal Procollagen III Propeptide (PIIINP) and Cytochrome b-245 Beta (CYBB, NOX2) levels, 3–4 mL of venous blood samples were collected in EDTA buffered tubes and centrifuged 10 min at 2000× *g* using a refrigerated centrifuge. Plasma samples were then stored at −80 °C. Plasma concentrations of PIIINP and NOX2 were measured by commercially available ELISA kits: Human N-Terminal Procollagen III Propeptide kit (Novus Biologicals, Centennial, CO, United States); Human Cytochrome b-245 Beta Polypeptide kit, (FineTest, Wuhan, China), according to the manufacturer’s instructions.

### 2.13. Statistical Analysis

For in vitro and animal study, Student’s *t*-test or one-way ANOVA were applied and values of *p*  <  0.05 were considered statistically significant. 

Normally distributed data were expressed in mean and standard deviation (SD). Non-normally distributed data were expressed in medians and interquartile ranges. Data were analyzed by using the intention to treat; in fact, the values recorded at baseline were compared with the values recorded at 4 months and after 24 months from the end of treatment in all patients, regardless of the treatment duration. Baseline and follow-up characteristics were tested for differences with the one-way ANOVA test (*p* < 0.05). The change in anthropometric and laboratory values, between placebo and treatment groups, was assessed by using repeated measures analysis of variance.

Pearson’s correlation test was used to evaluate a possible correlation between PIIINP, NOX2 and fibrosis.

GraphPad Prism 9.0 (GraphPad Software, San Diego, CA, USA), and MedCalc Software 19.2.6 (MedCalc Software, Ostend, Belgium) were used for statistics.

## 3. Results

### 3.1. The Combination Treatment with HXT and VitE Reduces TGF-β-Induced HSC Activation

The effect of different concentrations of HXT and VitE (ranging from 20 µM to 50 µM and 10 µM to 100 µM, respectively) on HSC LX-2 cell viability after 48 h supplementation of each compound, was first explored. As reported in Supplemental Figure S1, both HXT and VitE significantly increased LX-2 cells viability compared with non-treated (NT) cells. Therefore, based on these results, the 30 μM HXT and 20 μM VitE concentrations were chosen for subsequent experiments.

**Figure 1:**
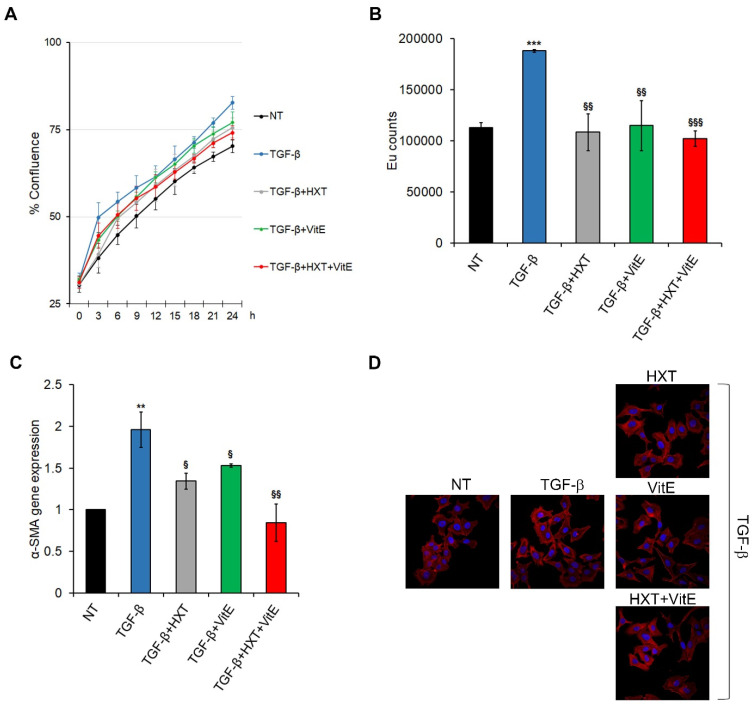
The combined treatment with HXT + VitE reduces the TGF-β-induced HSCs activation in LX-2 cells. (**A**) Real-time proliferation assay in LX-2 cells NT or treated with TGF-β alone or with indicated concentrations of HXT, VitE, or HXT + VitE for 24 h. Cell proliferation was reported as the percentage of confluence ± SD of two independent experiments. (**B**) Cell proliferation evaluated by a BrdU incorporation assay and expressed as Eu counts. Data are expressed as the mean ± SD of three independent experiments. *** *p* < 0.001 vs. NT; ^§§^ *p* < 0.01; and ^§§§^ *p* < 0.001 vs. TGF-β. (**C**) qRT-PCR of α-SMA in LX-2 cells NT or treated with TGF-β alone or with indicated concentrations of HXT, VitE, or HXT + VitE for 24 h. Data are expressed as the mean ± SD of three independent experiments. ** *p* < 0.01 vs. NT; ^§^ *p* < 0.05; and ^§§^ *p* < 0.01 vs. TGF-β. (**D**) Representative images of phalloidin (red) cellular localization by IF in LX-2 cells NT, or treated with TGF-β alone or with indicated concentrations of HXT, VitE, or HXT + VitE for 24 h. Nuclei were stained by Hoechst (blue). Magnification 40×.

The potential of the treatment with HXT and VitE to alter the TGF-β-induced activation of HSCs LX-2 cells, was investigated. TGF-β caused a significant increase of proliferation rate of LX-2 cells compared to the untreated counterpart, and the treatment with HXT and VitE, alone or in combination, induced a significant decrease of this TGF-β-dependent pro-proliferative effect (Figure 1A,B, and Supplemental Figure S2). However, the TGF-β-induced effect on HSCs was more efficiently inhibited by the combined treatment with HXT + VitE.

Next, the analysis of the consistent marker of HSC activation α-SMA, was performed. As shown in Figure 1C, TGF-β-induced upregulation of α-SMA gene expression was markedly reduced after 24 h treatment with HXT and VitE alone or in combination. The inhibitory effects of HXT + VitE on TGF-β-dependent activation of LX-2 cells was also confirmed by phalloidin staining (Figure 1D). These results indicate that the in vitro liver fibrosis model was successfully established and that the combined treatment HXT + VitE was more efficient in mitigating the TGF-β-induced HSCs activation. For this reason, the combined treatment was selected for the next experiments.

### 3.2. The Combination Treatment with HXT and VitE Inhibits TGF-β-Induced HSC Migration, Contractility and Pro-Fibrogenic Phenotype

In their activated phenotype, the upregulation of α-SMA expression in HSCs is accompanied by the acquisition of migratory and contractility properties with a consequential shift into a myofibroblast-like phenotype [22]. Here, we evaluated the effect of HXT + VitE on these pro-fibrogenic events that occurred in TGF-β-activated LX-2 cells. To this end, cells were pre-treated or not with TGF-β and then stimulated with HXT + VitE at different timepoints.

**Figure 2:**
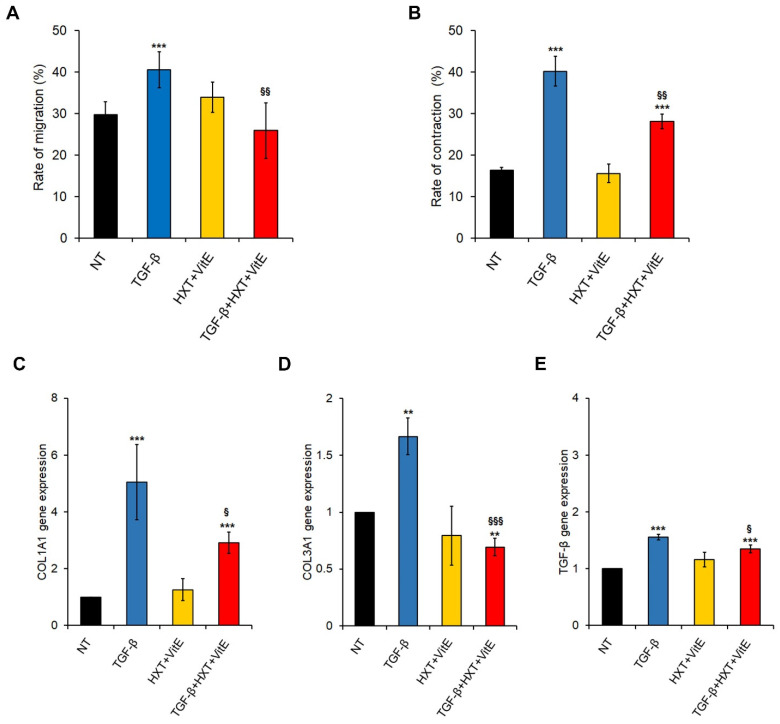
HXT + VitE attenuates TGF-β-induced myofibroblast-like and pro-fibrogenic phenotype in LX-2 cells. (**A**) Rate of migration by gap closure assay expressed as percentage in LX-2 cells NT, treated with HXT + VitE, or treated with TGF-β alone or with HXT + VitE at 0 and 12 h. Data are expressed as the mean ± SD of three independent experiments. *** *p* < 0.001 vs. NT; ^§§^ *p* < 0.01 vs. TGF-β. (**B**) Analysis of the three-dimensional gel contraction assay in LX-2 cells NT, treated with HXT + VitE, or treated with TGF-β alone or with HXT + VitE at 0 and 24 h. Data are expressed as the mean ± SD of three independent experiments. *** *p* < 0.001 vs. NT; ^§§^ *p* < 0.01 vs. TGF-β. Gene expression by qRT-PCR of COL1A1 (**C**), COL3A1 (**D**), and (**E**) TGF-β € in LX-2 cells NT, treated with HXT + VitE, or treated with TGF-β alone or with HXT + VitE for 24 h. Data are expressed as the mean ± SD of three independent experiments. ** *p* < 0.01 and *** *p* < 0.001 vs. NT; ^§^ *p* < 0.05; and ^§§§^ *p* < 0.001 vs. TGF-β.

First, the effect of HXT + VitE on migration after 12 h was examined by performing a gap closure assay. As expected, TGF-β-activated LX-2 cells migrated faster than NT cells, and the exposure of HXT + VitE exposure of TGF-β-treated cells significantly slowed the migration rate at levels similar to those observed in control cells (Figure 2A and Supplemental Figure S3A).

Next, the effect of HXT + VitE on cell contractility was evaluated after 24 h by employing a collagen gel contraction assay was evaluated. As shown in Figure 2B and Supplemental Figure S3B, the size of collagen gel with TGF-β stimulated HSCs became smaller than in all other experimental conditions, indicating that TGF-β promoted contractility of LX-2 cells. Interestingly, the TGF-β-promoted contraction rate resulted in attenuation by HXT + VitE, even if it didn’t reach the control levels.

Finally, the effect of HXT + VitE on the gene expression of fibrosis-related genes by qRT-PCR after 24 h were examined. As reported in Figure 2C–E the upregulation of COL1A1, COL3A1 and TGF-β mRNA levels observed after TGF-β stimulation was significantly counteracted by HXT + VitE treatment, reaching values near to controls for both COL3A1 and TGF-β.

### 3.3. The Combination Treatment with HXT and VitE Inhibits TGF-β-Induced HSC Oxidative Stress by Attenuating the TGF-β/SMAD Signaling Pathway

**Figure 3:**
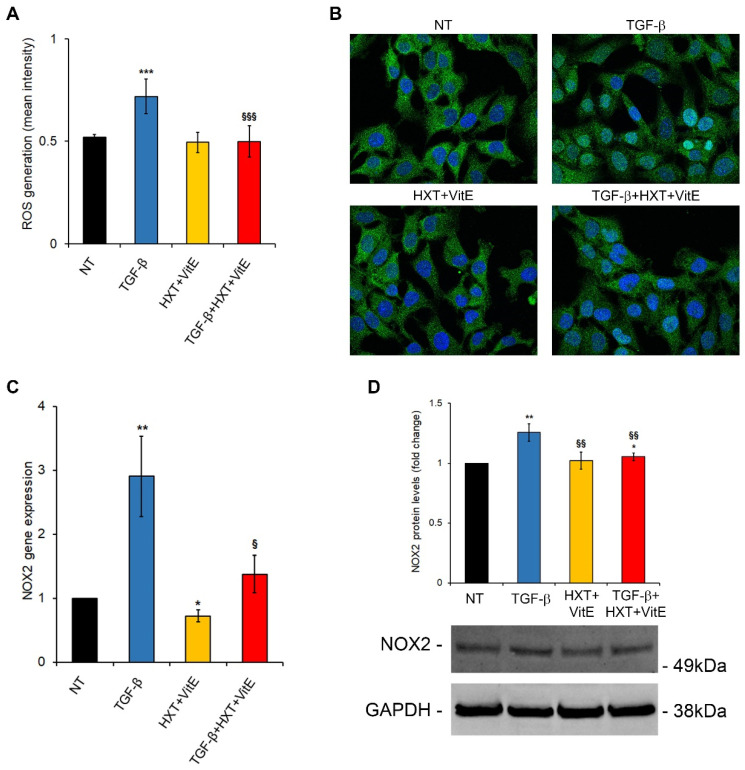
HXT + VitE improves TGF-β-pro-fibrogenic phenotype by attenuating the SMAD/NOX2 pathway. (**A**) ROS levels assessed by using CM-H_2_DCFDA in LX-2 cells NT, treated with HXT + VitE, or treated with TGF-β alone or with HXT + VitE for 24 h. Data are expressed as the mean ± SD of at least five independent experiments. *** *p* < 0.001 vs. NT; ^§§§^ *p* < 0.001 vs. TGF-β. (**B**) Representative images of SMAD2/3 (green) cellular localization by IF in LX-2 cells NT, treated with HXT + VitE, or treated with TGF-β alone or with HXT + VitE for 3 h. Nuclei were stained by Hoechst (blue). Magnification 60×. (**C**) NOX2 gene expression by qRT-PCR in LX-2 cells NT, treated with HXT + VitE, or treated with TGF-β alone or with HXT + VitE for 24 h. Data are expressed as the mean ± SD of three independent experiments. * *p* < 0.05 and ** *p* < 0.01 vs. NT; ^§^ *p* < 0.05 vs. TGF-β. (**D**) Quantitative analysis and representative immunoblot of NOX2 protein expression by WB in LX-2 cells NT, treated with HXT + VitE, or treated with TGF-β alone or with HXT + VitE for 24 h. GAPDH protein levels were used as loading control. Data are expressed as the mean ± SD of three independent experiments. * *p* < 0.05, ** *p* < 0.01 vs. NT; ^§§^ *p* < 0.01 vs. TGF-β.

Lines of evidence highlighted that TGF-β/SMAD signaling may play a major role in HSC activation by enhancing ROS generation and suppressing antioxidant enzymes, leading to a redox imbalance, cell damage, and fibrotic responses, thus promoting pro-fibrogenic processes in many organs, including the liver [23,24]. Here, the ability of the HXT + VitE combination to suppress the TGF-β induced oxidative stress was examined. It has been found that ROS were induced by TGF-β after 24 h of treatment, and that the HXT + VitE combination significantly decreased ROS levels, restoring them to control levels (Figure 3A). One of the signaling pathways appointed to sustain TGF-β related production of intracellular ROS is the nuclear translocation/activation of its downstream mediators SMAD2/SMAD3 and their related target genes, including NOXs, collagens and newly produced TGF-β, generating a perverse cycle of fibrogenesis [24,25]. Thus, the modulatory effect of HXT + VitE on this signaling pathway, was explored. As reported in Figure 3B, treatment with TGF-β treatment alone after 3 h caused a marked nuclear translocation of SMAD2/3 in LX-2 cells that was abolished by HXT + VitE combination. Next, if this impairment of SMAD2/3 nuclear translocation affected NOX2 gene and protein expression, was evaluated. As expected, TGF-β alone induced after 24 h a significant increase of NOX2 expression at mRNA and protein levels after 24 h (Figure 3C,D). It is noteworthy that HXT + VitE treatment was able to attenuate the TGF-β-induced upregulation of NOX2 mRNA and protein (Figure 3C,D). 

### 3.4. HXT + VitE Reduces CCl_4_ plus WD-Induced Liver Fibrosis in Mice

To confirm the anti-fibrogenic effect of the HXT + VitE combination therapy a model of NAFLD-related fibrosis by combining CCl_4_ and WD (WD), was established [26]. In particular, mice treated with CCl_4_ for 12 weeks were assigned to a treatment with WD or WD combined with HXT + VitE for an additional 2 weeks.

**Figure 4:**
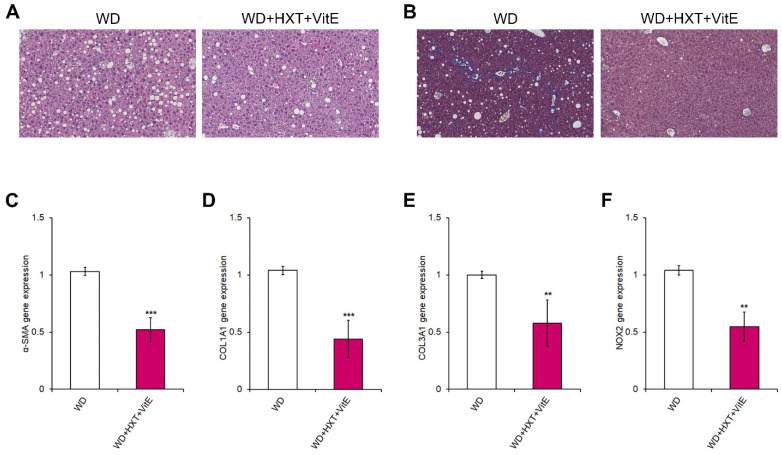
HXT + VitE improves WD-induced liver fibrosis in mice. Representative images of H&E (**A**) and Mason’s trichrome (**B**) staining in liver sections of WD and WD + HXT + VitE mice (20× magnification). Hepatic gene expression by qRT-PCR of α-SMA (**C**), COL1A1 (**D**), COL3A1 (**E**), and NOX2 (**F**) in WD and WD + HXT + VitE mice. Data are expressed as the mean ± SD of at least three independent experiments. ** *p* < 0.01 and *** *p* < 0.001 vs. WD.

WD mice exhibited a pattern of steatosis, inflammation and fibrosis that resembles human NASH, whereas, WD mice treated with HXT + VitE showed a marked decrease of both steatosis and fibrosis (Figure 4A,B).

Afterward, the expression levels of fibrosis-related genes in both groups of mice were explored, resulting in the discovery that the mRNA levels of α-SMA, COL1A1, and COL3A1 were reduced in WD-fed mice receiving HXT + VitE treatment compared to WD-fed mice receiving the vehicle alone (Figure 4C–E). Interestingly, in this in vivo model of NASH-related liver fibrosis the expression of NOX2 gene was significantly reduced in HXT + VitE-treated WD-fed mice compared to WD-fed control mice group (Figure 4F). 

### 3.5. HXT + VitE Treatment Reduces PIIINP and NOX2 Levels in Children with NAFLD

Anthropometrical, biochemical, and ultrasound data of the study population was reported in Table 1. In the PLA group, there is a significant worsening of basal fasting insulin values at T2. This effect was reflected in HOMA-IR value that resulted significantly increased in PLA group at T2 (*p* = 0.03). In the HXT+VitE group, there was a significant decrease from T0 to T2 of AST (*p* = 0.04), ALT (*p* = 0.05), GGT (*p* = 0.04), and triglycerides levels (*p* = 0.03). Also the number of patients with severe steatosis continued to be strongly reduced after treatment and follow-up only in HXT+VitE group.

Comparing the two groups at the end of the treatment (T1), there was a significant decrease of triglycerides (*p* = 0.04) and LDL levels (*p* = 0.04) in HXT+VitE group with respect to the PLA group. Furthermore, after 4-months, also the prevalence of severe steatosis decreased significantly in the HXT+VitE group compared to the PLA group, with a concomitant increase of patients with moderate steatosis. Interestingly, the improvement of both lipid profile and steatosis grade observed at T1 remained significant at follow-up (T2).

**Figure 5:**
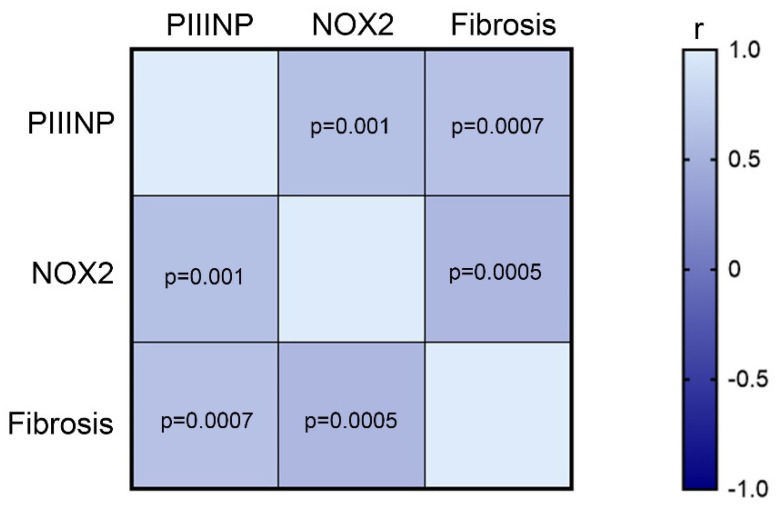
PIIINP and NOX2 levels correlate with fibrosis. Heatmap showing the correlation among the levels of PIIINP, NOX2, and fibrosis at T1 in patients included in our study.

Liver histology at T1 showed that 4 (16%) patients had no fibrosis, whereas 9 (36%) and 12 (48%) patients had fibrosis of stage 1 and stage 2, respectively. In these patients, the circulating levels of PIIINP and NOX2, were analyzed. As shown in Figure 5, the Pearson’s correlation analysis on all patients at T1 showed that the fibrosis positively correlated with PIIINP (r = 0.63, IC 95% 0.29 to 0.82, *p* = 0.0007), and with NOX2 levels (r = 0.64, IC 95% 0.32 to 0.83, *p* = 0.0005). Moreover, the levels of PIIINP and NOX2 showed a positive correlation (r = 0.61, IC 95% 0.27 to 0.81, *p* = 0.001).

**Figure 6:**
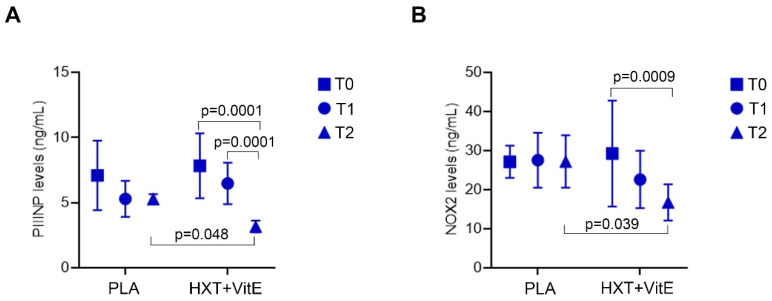
HXT + VitE treatment reduces the circulating levels of PIIINP and NOX2 in patients with NAFLD-related fibrosis. PIIINP (**A**) and NOX2 (**B**) circulating levels at T0, T1, and T2 in patients belonging to HXT + VitE or PLA group. Values are mean ± SD. The *p* values for the time-by-group interaction from repeated measures one-way ANOVA are shown.

Therefore, the changes of circulating levels of PIIINP and NOX2 during the treatments in our study population, were evaluated. As reported in Figure 6, our data revealed that PIIINP and NOX2 levels decreased significantly only after the treatment with HXT + VitE, and mainly at T2 compared to T0. Finally, at T2, the levels of both PIIINP and NOX2 were significantly lower in the HXT + VitE group than in PLA group of patients. 

## 4. Discussion

In this study, the potential therapeutic effect of HXT and VitE alone or in combination against NAFLD-related hepatic fibrosis using both in vitro and in vivo approaches, was evaluated.

In response to a variety of chronic stimuli, such as NAFLD, HSCs may acquire a pro-fibrogenic-activated phenotype, in which TGF-β acts as a pivotal driver. Once HSCs are activated, they undergo an increase of proliferation, migration, and contractility rates, thus leading to excessive deposition of extracellular matrix (ECM) proteins that substitute normal liver parenchyma with scar tissue [22]. Even though molecular and cellular mechanisms of liver fibrosis have been explored for a long time, nowadays there are no drugs specifically approved by the FDA or European Medicines Agency (EMA) for the treatment of fibrosis [27].

To date, promising therapeutic evidence recommends the use of specific EVOO-based dietary or nutraceutical interventions in metabolic diseases, such as NAFLD [28]. The interest of polyphenols extracted from EVOO as nutraceutical supplementation in NAFLD is magnified by studies demonstrating that these compounds may exert anti-fibrotic effects in both in vitro and in vivo models [29,30]. However, EVOOs contain also a relevant concentration of other bioactive compounds, such as VitE, which has been reported to improve hepatic fibrosis scores in patients with NASH [31]. Therefore, targeting the activation of HSCs by natural compounds, in single or in combination therapy, has becoming a promising interventional approach to reverse liver fibrosis [32].

**Figure 7:**
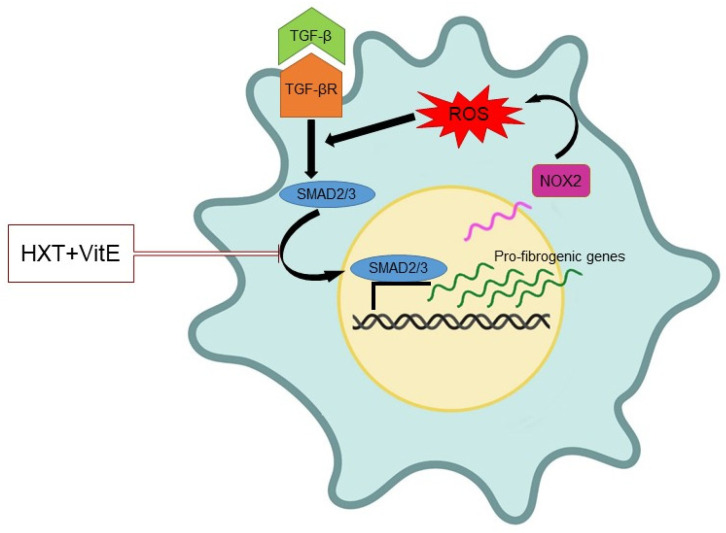
Schematic representation of the mechanism of action of HXT + VitE treatment in TGF-β-activated HSCs. Partially created with BioRender.com.

Results of the present study demonstrated that combination treatment with HXT + VitE may reduce the TGF-β-induced HSC activation. In particular, the exposure to HXT + VitE was effective in decreasing the gene expression levels of α-SMA, known as promoter of contractility in activated HSCs and consequent increase of ECM stiffness [33]. In addition, our data demonstrated that HXT + VitE affected proliferation and migration rate in TGF-β-activated HSCs. Overall, all these findings suggest that the combined treatment with the two natural compounds is able to counteract the HSC acquisition of a pro-fibrogenic phenotype. Because both HXT and VitE are well known for their antioxidant properties, it is possible to speculate that the impairment of oxidative stress and related signaling pathways could be the key mechanisms underlining the observed reversion of myofibroblast-like pattern in HSCs [34]. Accordingly, it has been observed that HXT + VitE combined treatment may block TGF-β-induced ROS accumulation in association with the impairment of the nuclear translocation/activity of SMAD2/3 transcription factors, and expression of their target genes, including COL1A1, COL3A1, NOX2, and TGF-β, thus interrupting the self-sustained circle of fibrosis (Figure 7).

The in vitro findings were confirmed by a pilot study conducted on a mice model of NAFLD-related fibrosis. Indeed, we found that HXT + VitE decreased both hepatic damage and fibrosis-related gene expression levels. Even if these data seem to be preliminary, they reinforce also the previous beneficial effects of combined therapy with HXT and VitE observed in pediatric NAFLD [18,19]. In the aforementioned randomized trial, data showing the anti-steatotic, antioxidant, and anti-inflammatory effects of 4-month-long therapy with a combination of HXT and VitE compared to a placebo group, were reported. However, in this previous clinical study the potential effect on fibrosis was not evaluated because histological data on fibrosis was available only at baseline. Fortunately, during the last several years the intense research focused on attempting to reduce liver biopsy use for diagnosis and follow-up of fibrosis in patients with NAFLD, has produced several useful non-invasive circulating biomarkers for advanced fibrosis and active fibrogenesis, such as PIIINP [35]. Interestingly, in a clinical study involving children and adolescents with biopsy-proven NAFLD, the measure of circulating levels of PIIINP has revealed its accurate diagnostic performance for fibrosis staging [36]. Moreover, the soluble NOX2-derived peptide (sNOX2-dp), a direct marker of NADPH oxidase activation, was found to be significantly increased in children with biopsy-proven NAFLD compared to control subjects and significantly correlated with the degree of liver damage and oxidative stress [37]. Hence, a study for the evaluation of the circulating levels of PIIINP and NOX2, as potential non-invasive biomarkers of fibrosis in 25 NAFLD children enrolled in the 4-month interventional study with HXT-VitE and who participated to a long-term follow-up study (24 months from the end of treatment), were designed. In line with the literature evidence [36,37], in the study participants it has been observed that PIIINP and NOX2 levels positively correlated with fibrosis at baseline. Interestingly, the levels of these two biomarkers decreased only in the treatment group after 4 months, and this trend of reduction is maintained as also being significant during follow-up. Consequently, at the end of the 24-month follow-up study, participants that had received HXT + VitE combination therapy exhibited lower levels of circulating PIIINP and NOX2 when compared to the placebo group. These last findings strongly confirm our hypothesis, suggesting that short-term therapy with HXT + VitE may reduce fibrosis stage in pediatric NAFLD, and that this beneficial effect is supported over time. 

## 5. Conclusions

Even though our study is the first evidence of the beneficial effects of HXT + VitE on NAFLD-related fibrosis, it has three major limitations. The first limitation is that data obtained in mice are the result of a pilot study performed in a single model of NAFLD-related fibrosis. The other limitations are associated to the study in humans: i) due to the invasive nature of liver biopsy, the level of PIIINP and NOX2 with fibrosis staging at follow-up cannot be correlated among them; and ii) due to COVID-19 pandemic restriction, only a small number of patients accepted the offer to participate to the follow-up study.

In an effort to overcome such limitations and better explore the mechanisms underlining HXT + VitE effects on fibrosis, new more rigorous clinical studies and further experimental investigation are highly desirable in the next future.

In conclusion, the present study demonstrated that supplementation with HXT and VitE can be a potential therapeutic approach to improve NAFLD-related liver fibrosis, thus reducing the risk of disease progression.

## Figures and Tables

**Table 1:** Patients’ characteristics.

	PLA Arm (*n* = 9)				HXT + VitE Arm (*n* = 16)				PLA vs. HXT + VitE (T1)	PLA vs. HXT + VitE (T2)
	T0	T1	T2	*p*	T0	T1	T2	*p*	*p*	*p*
*Age (years)*	11.5 (2.6)	12.1 (2.6)	13.8 (2.6)	0.45	12.3 (2.4)	12.7 (2.4)	14.3 (2.3)	0.32	0.84	0.12
*BMI (Kg/m^2^)*	27 (6.7)	26.9 (6.9)	27.8 (3.6)	0.78	26.9 (4.2)	27.4 (4.9)	26.2 (3)	0.87	0.77	0.68
*WC (cm)*	92.3 (18.4)	98.2 (19.6)	94.2 (10.5)	0.56	94.3 (10.8)	98.1 (9.7)	95.8 (7.4)	0.94	0.92	0.84
*Total Cholesterol (mg/dL)*	172.5 (26.9)	172.5 (37.8)	161 (32.5)	0.12	161.5 (30.8)	149 (27.6)	157.4 (39.7)	0.51	0.10	0.42
*HDL-C (mg/dL)*	45.2 (5.9)	42.2 (5.7)	41.2 (6.4)	0.83	47.3 (6.5)	45.6 (7.2)	49 (9.8)	0.75	0.67	0.11
*LDL-C (mg/dL)*	117.5 (29.6)	117 (38.2)	106.3 (33.8)	0.07	101.4 (30.8)	95 (21)	95.1 (35.8)	0.27	**0.04**	**0.034**
*Triglycerides (mg/dL)*	112.3 (41.8)	167.3 (42.4)	117 (55)	0.08	115.7 (56.5)	87.7 (33.6)	90.2 (42.6)	**0.03**	**0.04**	**0.03**
*Uric Acid (mg/dL)*	5.5 (1.5)	5.9 (1.6)	6.5 (1.7)	0.11	6.1 (1.2)	5.9 (1.1)	6.4 (1.5)	0.40	0.96	0.95
*Fasting Glucose (mg/dL)*	87.2 (7.7)	84.7 (3.2)	89.7 (5.4)	0.66	86.5 (5.8)	86.7 (6.3)	91 (6)	0.71	0.67	0.57
*Fasting Insulin (mU/L)*	18.2 (2.8)	14.8 (3.2)	27.5 (15.4)	**0.05**	24.3 (8.8)	19.2 (10)	25.4 (10.7)	0.08	0.18	0.41
*HOMA- IR*	3.9 (0.7)	3.1 (0.7)	6.1 (3.5)	**0.03**	5.2 (2)	3.8 (2.3)	5.6 (2.2)	0.09	0.84	0.69
*AST (UI/L)*	29.4 (6.7)	28.8 (7.1)	28 (16.2)	0.74	44.4 (45.4)	36.7 (23.8)	24.7 (10.9)	**0.04**	0.11	0.22
*ALT (UI/L)*	40.3 (23.8)	39.1 (19.1)	35.2 (20.6)	0.24	59.9 (56.4)	45.2 (32.9)	34.6 (24.5)	**0.05**	0.57	0.89
*GGT (UI/L)*	20 (15.3)	19.4 (11.7)	26.4 (17)	0.21	27.6 (28)	23.1 (24.7)	21.6 (11.2)	**0.04**	0.61	0.46
*STEATOSIS*										
*MILD*	0	1 (11.1)	0	--	0	3 (18.7)	3 (18.7)	--	0.57	--
*MODERATE*	3 (33.3)	3 (33.3)	5 (55.5)	0.06	9 (56.2)	11 (68.8)	12 (75)	0.07	**0.02**	0.12
*SEVERE*	6 (66.7)	5 (55.6)	4 (44.5)	0.06	7 (43.8)	2 (12.5)	1 (6.3)	**0.001**	**0.03**	**0.001**

BMI, body mass index; WC, waist circumference; HDL-C, high-density lipoprotein-cholesterol; HDL-C, low-density lipoprotein cholesterol; HOMA-IR, homeostatic model assessment for IR; ALT, alanine aminotransferase; AST, aspartate aminotransferase; GGT, gamma-glutamyl transferase. Data are presented as mean and SD or medians and interquartile ranges Or number and percentage. *p*-values were calculated by using one-way ANOVA. Significant values are reported in bold.

## Data Availability

Not applicable.

## Supplementary Materials

The following supporting information can be downloaded at: https://www.mdpi.com/article/10.3390/nu14183791/s1. Figure S1, Treatment with HXT and VitE increases cell viability in LX-2 cells; Figure S2, Live cell imaging with the mask of cells; Figure S3, Effects of treatments on LX-2 cells migration and contraction rate.
