# Supplementary material for: Combination Treatment with Hydroxytyrosol and Vitamin E Improves NAFLD-Related Fibrosis
Source: Nutrients. 2022 Sep 14;14(18):3791. doi: 10.3390/nu14183791 (PMC9505330; doi:10.3390/nu14183791)
Supplement: Supplementary file 1 [file nutrients-14-03791-s001.zip › nutrients-1887317-supplementary.pdf]

Supplementary Data

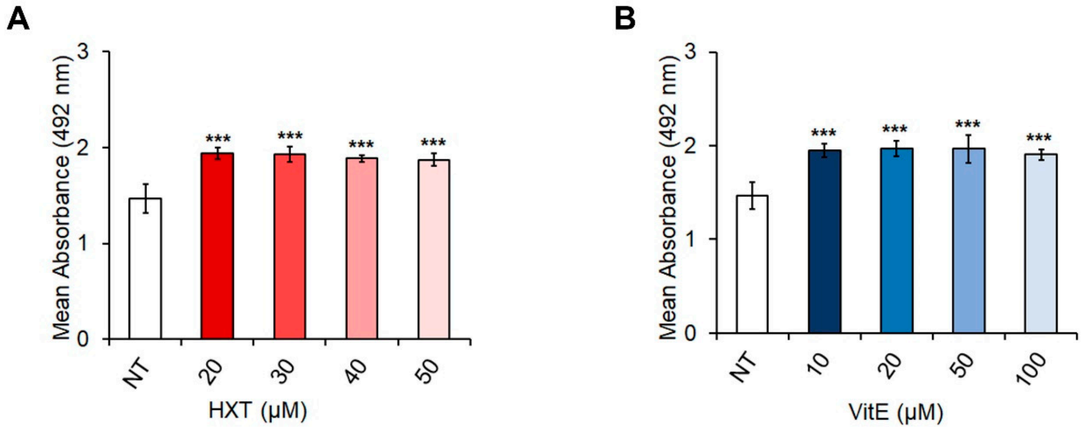

Figure S1. Treatment with HXT and VitE increases cell viability in LX-2 cells. Cell viability in LX-2 cells was assessed by XTT assay after 48 h of treatment with HXT (20, 30, 40, and 50 µM) or VitE (10, 20, 50, and 100 µM). Data are the mean absorbance of two independent experiments repeated at least in triplicate. Data were analyzed by 2-tailed t tests, ***p <0.001 vs NT.

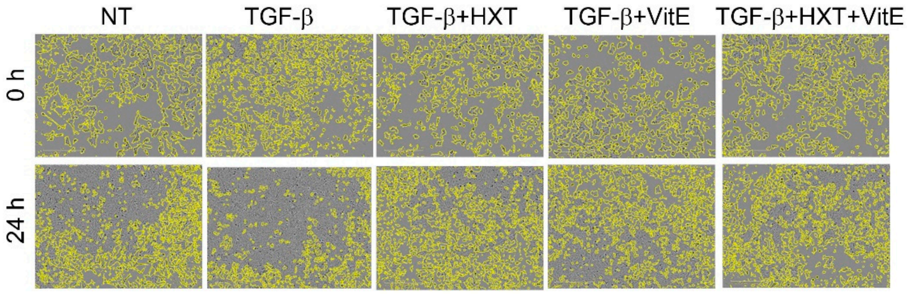

Figure S2. Live cell imaging with the mask of cells. Representative images of automatically identified regions of interest using confluence masks (empty areas are represented in yellow) of LX-2 cells NT or treated with TGF-β alone or with indicated concentrations of HXT, VitE, or HXT+VitE at 0 and 24 h.

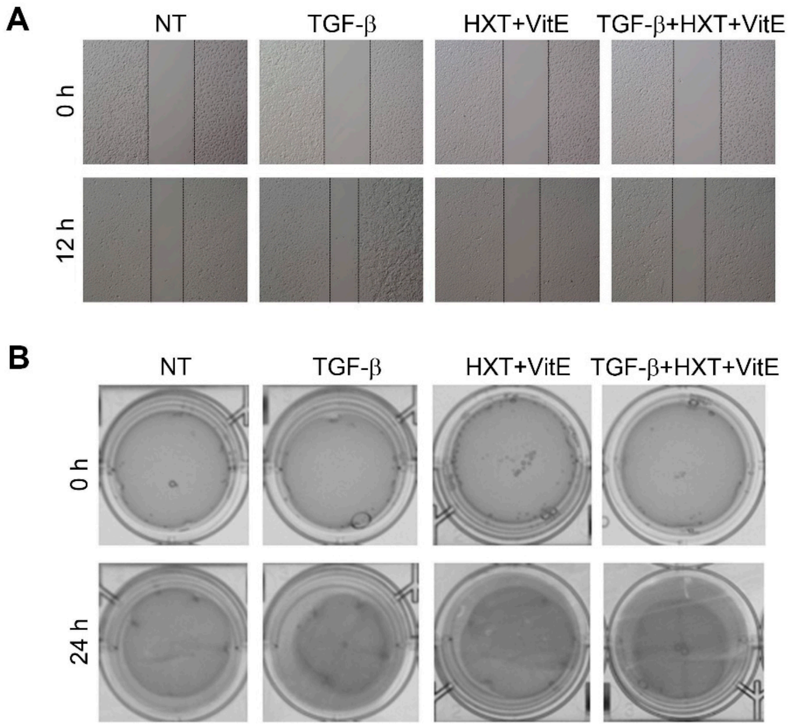

Figure S3. Effects of treatments on LX-2 cells migration and contraction rate. (**A**) Representative photographs of migration assay in LX-2 cells NT, treated with HXT+VitE, or treated with TGF-β alone or with HXT+VitE at 0 and 12 h. (**B**) Representative photographs of contraction assay in LX-2 cells NT, treated with HXT+VitE, or treated with TGF-β alone or with HXT+VitE at 0 and 24 h.
